# Surviving anoxia in marine sediments: The metabolic response of ubiquitous benthic foraminifera (*Ammonia tepida*)

**DOI:** 10.1371/journal.pone.0177604

**Published:** 2017-05-31

**Authors:** Charlotte LeKieffre, Jorge E. Spangenberg, Guillaume Mabilleau, Stéphane Escrig, Anders Meibom, Emmanuelle Geslin

**Affiliations:** 1 Laboratory for Biological Geochemistry, School of Architecture, Civil and Environmental Engineering (ENAC), Ecole Polytechnique Fédérale de Lausanne (EPFL), Lausanne, Switzerland; 2 Stable Isotope and Organic Geochemistry Laboratories, Institute of Earth Surface Dynamics (IDYST), University of Lausanne, Lausanne, Switzerland; 3 Service commun d'imageries et d'analyses microscopiques (SCIAM), Institut de Biologie en Santé, University of Angers, Angers, France; 4 Center for Advanced Surface Analysis, Institute of Earth Sciences, University of Lausanne, Lausanne, Switzerland; 5 UMR CNRS 6112 - LPG-BIAF, University of Angers, Angers, France; University of Southern Denmark, DENMARK

## Abstract

High input of organic carbon and/or slowly renewing bottom waters frequently create periods with low dissolved oxygen concentrations on continental shelves and in coastal areas; such events can have strong impacts on benthic ecosystems. Among the meiofauna living in these environments, benthic foraminifera are often the most tolerant to low oxygen levels. Indeed, some species are able to survive complete anoxia for weeks to months. One known mechanism for this, observed in several species, is denitrification. For other species, a state of highly reduced metabolism, essentially a state of dormancy, has been proposed but never demonstrated. Here, we combined a 4 weeks feeding experiment, using ^13^C-enriched diatom biofilm, with correlated TEM and NanoSIMS imaging, plus bulk analysis of concentration and stable carbon isotopic composition of total organic matter and individual fatty acids, to study metabolic differences in the intertidal species *Ammonia tepida* exposed to oxic and anoxic conditions. Strongly contrasting cellular-level dynamics of ingestion and transfer of the ingested biofilm components were observed between the two conditions. Under oxic conditions, within a few days, intact diatoms were ingested, degraded, and their components assimilated, in part for biosynthesis of different cellular components: ^13^C-labeled lipid droplets formed after a few days and were subsequently lost (partially) through respiration. In contrast, in anoxia, fewer diatoms were initially ingested and these were not assimilated or metabolized further, but remained visible within the foraminiferal cytoplasm even after 4 weeks. Under oxic conditions, compound specific ^13^C analyses showed substantial *de novo* synthesis by the foraminifera of specific polyunsaturated fatty acids (PUFAs), such as 20:4(*n*-6). Very limited PUFA synthesis was observed under anoxia. Together, our results show that anoxia induced a greatly reduced rate of heterotrophic metabolism in *Ammonia tepida* on a time scale of less than 24 hours, these observations are consistent with a state of dormancy.

## Introduction

Benthic foraminifera are eukaryote unicellular protists and ubiquitous in marine sediments from shallow water estuaries to the deep ocean [1]. Representing up to 50% of top sediment biomass, they constitute an important part of benthic meiofauna [2,3] and may play a significant role in the carbon and nitrogen cycles, depending on the habitat, species assemblage, and feeding patterns [4–7]. The broad spectrum of conditions under which marine foraminifera live includes zones of O_2_-depletion [8–11], deep-sea sulphidic habitats [12], hydrocarbon seeps [13,14], and intertidal environments [15]. Of particular interest here is the striking capability of some benthic foraminifera to adapt to a sudden decrease in the availability of O_2_. Hypoxic and anoxic events strongly and more frequently affect benthic ecosystems, in particular on continental shelves and in coastal areas where renewal of bottom water is slow and/or organic input is high [16–18]. During such events, large fractions of the benthic meio- and macrofauna (size range >1 mm) can die off [19–22]. However, foraminifera are consistently among the most resistant species [9,23,24]. High survival rates of foraminifera under low O_2_ conditions have been documented both *in-situ* [25–29] and in laboratory experiments [30,31] and ascribed, in part, to relatively low rates of O_2_-respiration compared to other meiofauna species [32]. Experimental studies of *Ammonia* sp. combining TEM and NanoSIMS observations suggest higher global metabolic activity in hypoxia than in anoxia [33]. Various anaerobic pathways have been suggested as alternative metabolic strategies to achieve resistance to low-O_2_ conditions, including symbiosis with ectobionts [12,34] or endobionts [35], and sequestered chloroplasts [36,37]. It has been demonstrated that some species are capable of nitrate respiration (denitrification) under anoxia [38–40]. Bernard et al. [41] observed a decrease of the adenosine 5′-triphosphate (ATP) pool in foraminifera *Bulimina marginata*, *Stainforthia fusiformis* and *Adercotryma glomeratum* from Drammensfjord (Norway) exposed to anoxia, and suggested that this might indicate a state of dormancy. Indeed, dormancy or quiescence, defined as reduced or suspended metabolic activity in response to exogenous factors, might be a more widespread adaptation strategy of benthic foraminifera to environmental stress than previously acknowledged [42]. Even during periods with normal oxic conditions in bottom waters, foraminifera and other benthic meiofauna species can be (and frequently are) exposed to low O_2_ levels simply because bioturbation mechanically moves them deeper into the sediments [43,44]. *Ammonia tepida*, for example, which is among the most abundant species in intertidal sediments [15] is normally residing in the top few centimeters of the sediments, where O_2_ concentration is high. Here, it grazes on algal biofilm [45]. However, *A*. *tepida* is also regularly found alive at depths of 4 to 26 cm, *i*.*e*. below the O_2_ penetration depth, as a result of bioturbation [46,47]. These observations raise questions about the mechanism(s) that enable foraminifera to survive sudden changes to anoxia, often for extended periods of time.

In this study, we present results of two experiments: Experiment I aimed to determine the survival and growth rates of algae-fed *A*. *tepida* under anoxia, compared with oxic conditions. Experiment II aimed to investigate the metabolism of *A*. *tepida* following a sudden shift to anoxic conditions. In the latter experiment, using ^13^C-enriched diatom-containing biofilm and a combination of transmission electron microscopy (TEM) and NanoSIMS isotopic imaging, we have visualized and quantified with subcellular resolution (*in situ*, *ex vivo*) the incorporation and transfer of isotopically labeled heterotrophic compounds, under both oxic and anoxic conditions. These subcellular-level observations were combined with concentrations and stable carbon isotopic analysis by isotope ratio mass spectrometry of total organic carbon (TOC) and individual fatty acids. Our results are discussed in context of previous experiments using ^13^C-labeled food, which have already yielded important insights into the metabolism of foraminifera under a variety of environmental conditions [48–55].

## Results

### Experiment I: Survival and growth rate of A. tepida under oxic and anoxic conditions

After 13 days of incubation, the survival rates of fed adult or juvenile specimens of *A*. *tepida* were indistinguishable (*p*>0.05) between oxic and anoxic conditions (S1 Fig): 95±11% and 87±12% for adults and 84±2% and 83±8% for juveniles, respectively. The average growth rate of juvenile specimens was significantly (*p*<0.05) higher under oxic (1.3±0.7% per day) compared with anoxic (0.2±0.1% per day) conditions (S2 Fig). The growth rate under anoxic conditions was not significantly different from zero (*t*-test, *p*>0.05).

### Experiment II: Feeding behavior of A. tepida under oxic and anoxic conditions

Under anoxic conditions the foraminifera rapidly (within around 24 hours) ceased to move. At the end of the incubation there was still diatom biofilm left in the vials in the anoxic aquarium, while the biofilm had been completely consumed by the foraminifera in the oxic aquarium.

**Fig 1:**
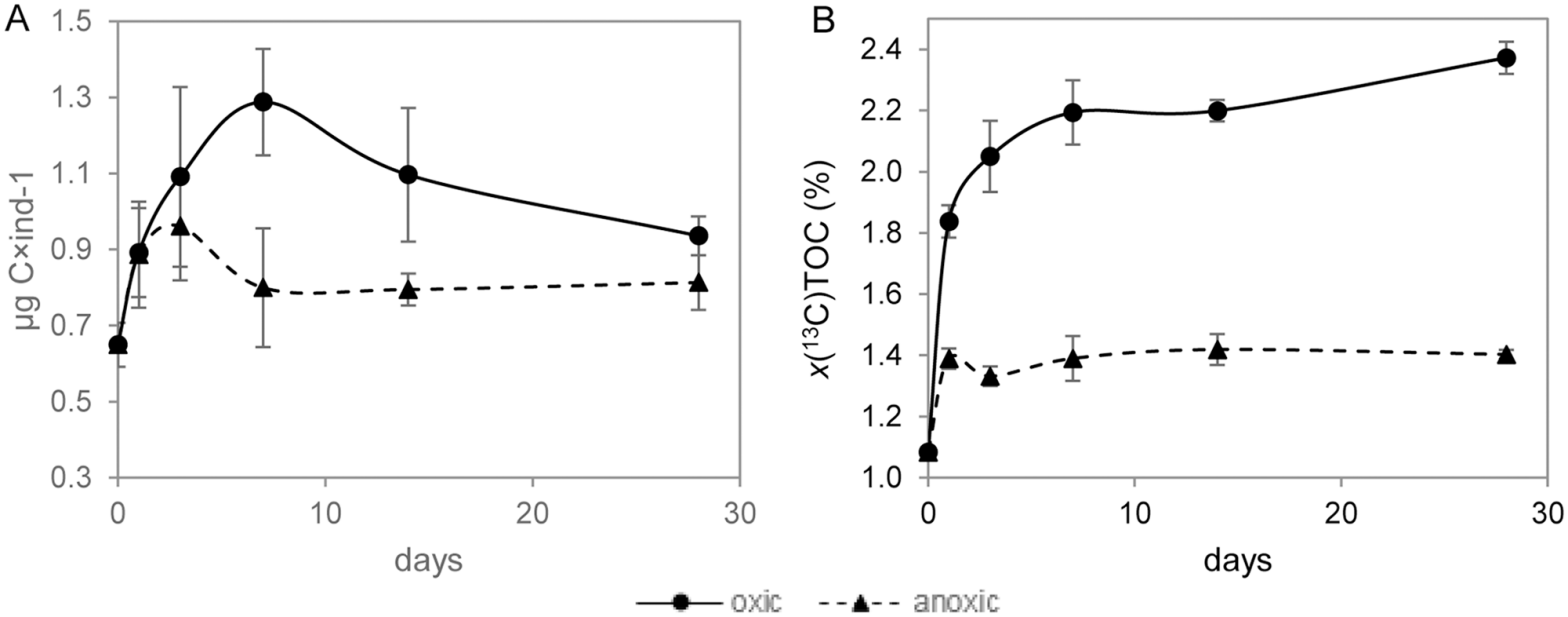
TOC concentration and ^13^C atomic fraction of *A*. *tepida* under oxic and anoxic conditions. (A) Average total organic carbon (TOC in μg C×ind^-1^) concentration and (B) ^13^C atomic fraction of the TOC (*x*(^13^C)_TOC_ in %), both as a function of time. Continuous lines: oxic conditions, dotted lines: anoxic conditions. Error bars are ±1 SD (*n* = 3).

Under oxic conditions, the average total organic carbon (TOC) content per cell of the *A*. *tepida* specimens increased during the first 7 days from 0.65±0.06 to 1.29±0.14 μg C×ind^-1^ (Fig 1A). At this point it was observed that all the biofilm had been ingested. After 14 days, the TOC content had decreased to 1.10±0.18 μg C×ind^-1^ and continued to decrease to reach a value of 0.94±0.05 μg C×ind^-1^ at the end of the experiment (*i*.*e*. Day 28). Under anoxia the TOC content showed a modest increase during the first 3 days of the incubation, reaching maxima of 1.0±0.1 μg C×ind^-1^. At Day 7, the TOC had dropped to 0.8 ±0.1 μg C×ind^-1^ and this level was maintained for the rest of the experiment (*p*>0.05) (Fig 1A).

Average ^13^C atomic fractions in TOC (*x*(^13^C)_TOC_ in %) as a function of time are shown in Fig 1. Under both oxic and anoxic conditions, a sharp ^13^C-enrichment indicating an uptake of ^13^C-enriched diatoms occurred at the beginning of the experiment, reaching plateaus on different time scales. Under oxic conditions, a sharp increase in *x*(^13^C)_TOC_ up to 1.86±1.16% occurred during the Day 1, followed by a slower increase to 2.24±1.22% on Day 7, after which *x*(^13^C)_TOC_ stabilized (*p*>0.05). Under anoxia, *x*(^13^C)_TOC_ increased to 1.41±1.18% during the Day 1, after which no statistically significant changes were observed (*p* >0.05). The final ^13^C_TOC_-enrichment was about 4 times higher under oxic than anoxic conditions.

Average carbonate uptake (calculated as the difference in C content of the shells in individuals from Day 28 and control specimens) and the enrichment in ^13^C of the shells from Day 28 over that of control samples are given in S1 Table. Under oxic conditions, an average of 4.9±1.8 μg C×ind^-1^ was added to the shells over 28 days and their average *x*(^13^C)_car_ was 0.05% higher than the unlabeled control samples. Under anoxia, the specimens did not add new carbonate to their shells and therefore no significant ^13^C_car_-enrichment was observed (*p*>0.05), consistent with a growth rate statistically indistinguishable from zero (Fig 1B).

**Fig 2:**
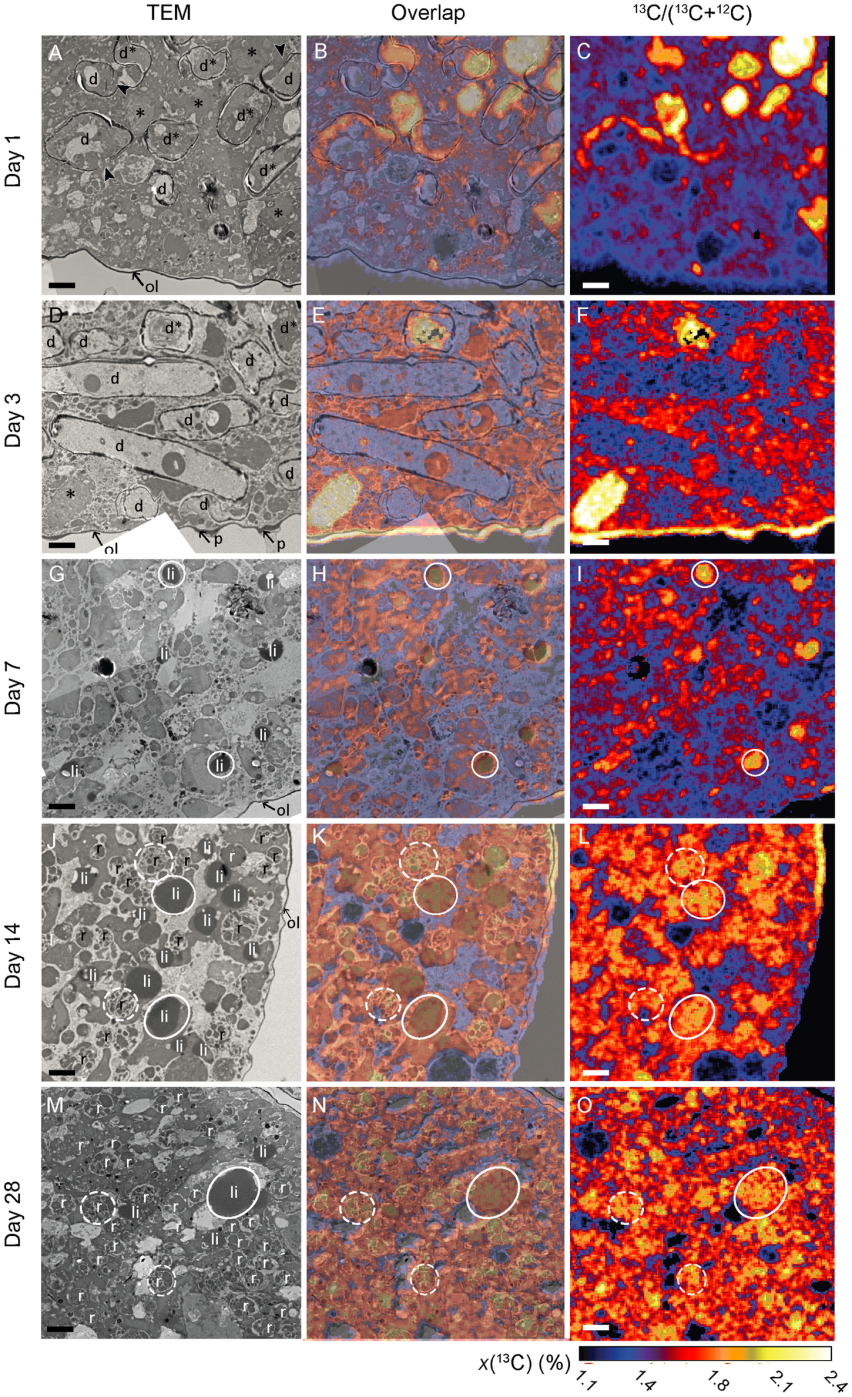
Time-evolution of ^13^C uptake and transfer within the cytoplasm of *A*. *tepida* under oxic conditions. A, D, G, J and M: TEM images; C, F, I, L and O: NanoSIMS images of corresponding ^13^C/^12^C distributions. B, E, H, K, and N: Direct correlation of TEM and NanoSIMS images. d*: Intact diatoms; d: frustules without their original contents; *: diatomic material free in the foraminiferal cytoplasm; li: lipid droplets; ol: organic lining; p: pores; r: residual bodies. Arrowheads show aperture of opened diatom frustules. Circles are drawn around a few organelles to facilitate their visualization on the different images: white circles: lipid droplets, dotted circles: residual bodies. Scale bars: 2 μm.

**Fig 3:**
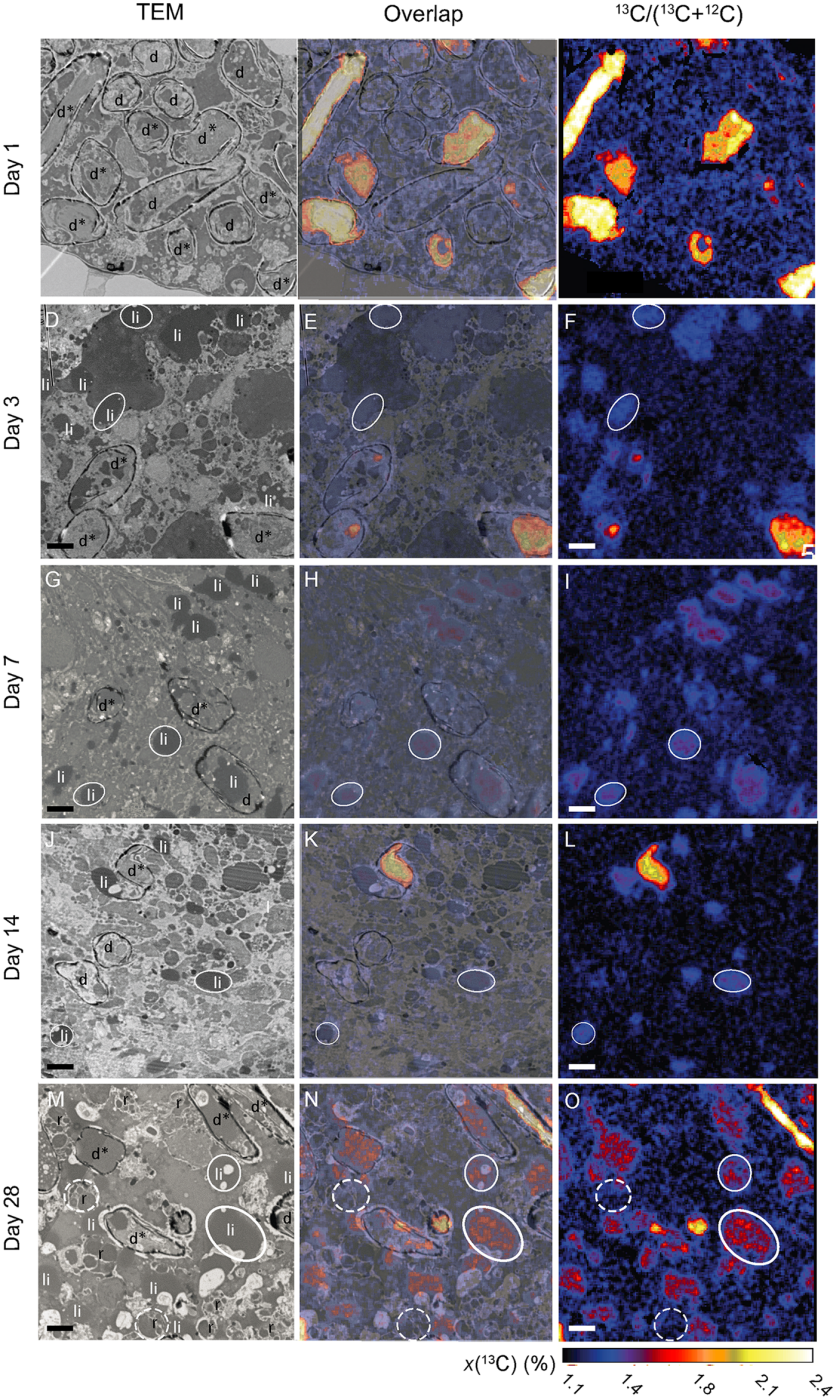
Time-evolution of ^13^C uptake and transfer within the cytoplasm of *A*. *tepida* under anoxic conditions. A, D, G, J and M: TEM images; C, F, I, L and O: NanoSIMS images of corresponding ^13^C/^12^C distributions. B, E, H, K, and N: Direct correlation of TEM and NanoSIMS images d*: Intact diatoms; d: frustules without their original contents; *: diatomic material free in the foraminiferal cytoplasm; li: lipid droplets; ol: organic lining; p: pores; r: residual bodies. Arrowheadsshow aperture of opened diatom frustules. Circles are drawn around a few organelles to facilitate their visualization on the different images: white circles: lipid droplets, dotted circles: residual bodies. Scale bars: 2 μm.

**Fig 4:**
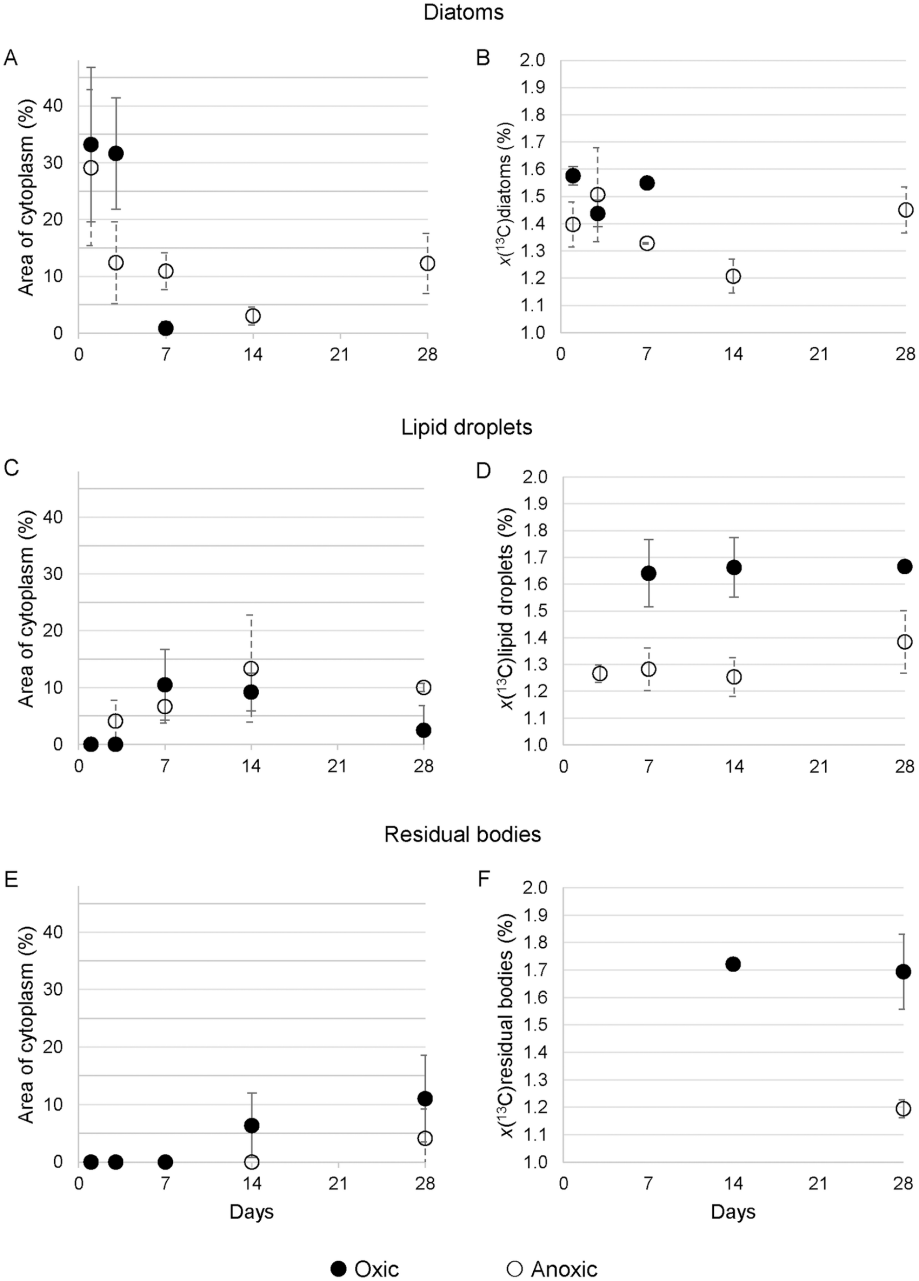
Percentages of cytoplasmic occupation and ^13^C atomic fraction of key cell ultrastructures. Percentage of occupation of cytoplasm area (A, C and E) and ^13^C atomic fraction (*x*(^13^C) in %; B, D and F) over time for key components in *A*. *tepida*: A, B: diatoms; C, D: lipid droplets; E, F: residual bodies. Errors bars are ±1 SD (*n* = 3).

Results of TEM and NanoSIMS analyses are presented in Figs 2 and 3. S3 Fig exhibits typical cellular structures in the antepenultimate chamber of an *A*. *tepida* specimen collected directly from the mudflat that provided samples for Experiment II. Recognizable structures include lipid droplets, residual bodies, and diatomic frustules. The presence of mitochondria and the integrity of intact double membranes and crests indicated vitality at the time of the chemical fixation; all observed specimens exhibited these ultrastructures. Time sequences of TEM and NanoSIMS isotopic images permit to follow the ingestion and metabolism of isotopically enriched diatom biofilm components under oxic and anoxic conditions (Figs 2 and 3). Fig 4 shows the relative surface areas occupied by diatoms, lipid droplets, and residual bodies in a representative cytoplasm area, with the corresponding average ^13^C atomic fractions for each structural component.

Under oxic conditions, abundant diatoms with frustules were visible after Day 1 as free-floating objects (*i*.*e*. not surrounded by vesicles/vacuoles) that occupied about 30% of the cytoplasm area (Figs 2A and 4A). About 75% of these ingested diatoms still held their original cellular matrix, which was clearly distinguishable by strong ^13^C enrichment; the remaining 25% had lost their content to the foraminiferal cytoplasm (Fig 2A–2C, Table 1). After Day 3, diatom frustules were still clearly observable (Fig 2D), but ca. 83% of them had lost their original content of cellular matrix (Fig 2D–2F, Table 1). After Day 7, frustules were no longer observed (Fig 2G, 2J and 2M). However ^13^C-enriched lipid droplets (not observed before Day 7) were numerous (Fig 4C). Between Day 7 and 14, lipid droplets were present in roughly constant abundance (ca. 10%; Fig 4C) with *x*(^13^C) of approximately 1.65% (Fig 4D). After Day 28 only a few lipid droplets were observed in the cytoplasm of the foraminifera (Figs 2M and 4C). In contrast, ^13^C-enriched residual bodies appeared after Day 14 (Figs 2J and 4F) occupying about 5% of the cytoplasm area with an average ^13^C atomic fraction around 1.70% (Fig 4E and 4F); this did not significantly change before the end of the experiment (*p*>0.05). In 5 out of 15 observed foraminifera cells, the organic lining (*i*.*e*. the thick membrane between the plasma membrane and the calcite shell) was enriched in ^13^C (Fig 2E, 2F, 2K and 2L); two of these had the ^13^C-enrichment of their organic lining concentrated in the vicinity of pores in the shell.

**Table 1:** Percentage of intact diatoms (frustule containing cytoplasm) in the foraminiferal cytoplasm.

Days	Diatoms filled with diatomic material (%)	
	Oxic	Anoxic
1	75 ±11	91 ±8
3	17 ±10	73 ±22
7	0	28 ±22
14	0	17^a^
28	0	47 ±46

^a^: diatoms were present only in 1 of the 3 specimens analyzed, SD could not be calculated.

Percentage of the diatoms present in the cytoplasm of *A*. *tepida* still holding their original cellular contents, as a function of time for both experimental conditions (*n* = 3).

Under anoxic conditions, the content of the foraminifera cytoplasm after Day 1 was essentially identical to that observed at the same time under oxic conditions (Fig 3A). No lipid droplets or residual bodies were visible, and the cytoplasm was occupied by ^13^C-labeled intact diatoms (*i*.*e*. diatomic material surrounded by the silica frustule; roughly 30% of the imaged area) (Figs 3A–3C and 4A–4B). However, the fraction of ingested diatoms still containing their original ^13^C-labeled material was higher under anoxic conditions (roughly 91% vs. 75%; Table 1). After Day 3 diatoms were still observed in the cytoplasm (Fig 3D) with about 75% of them containing original cellular materials; *i*.*e*., 4 times more than under oxic conditions at the same time point (Table 1). The proportion of diatoms in the foraminifera cytoplasm remained roughly constant between Days 3 and 28, in the range from 3 to 12% (Fig 4A). Among these, the proportion containing original ^13^C-labeled material decreased to ca. 30% between Days 3 and 7, and then did not significantly change until the end of the experiment (*p*>0.05; Table 1). Lipid droplets appeared after Day 3 under anoxic conditions, in contrast to Day 7 under oxic conditions (Fig 3D and 3C). Their proportion in the cytoplasm varied between 4% and 19% with corresponding average ^13^C atomic fractions between 1.27% and 1.38%; *i*.*e*. 2 to 3 times less ^13^C-enrichment than under oxic conditions (Fig 4C and 4D). Residual bodies, which were observed only in specimens sampled on Day 28, and only in 2 out of 3 imaged foraminifera, were much less abundant (4±4%) than under oxic conditions (Figs 3M and 4E). Most of these residual bodies were only slightly enriched, with an average *x*(^13^C) of 1.20±0.03% compared to 1.69±0.14% under oxic conditions (Fig 4F).

**Fig 5:**
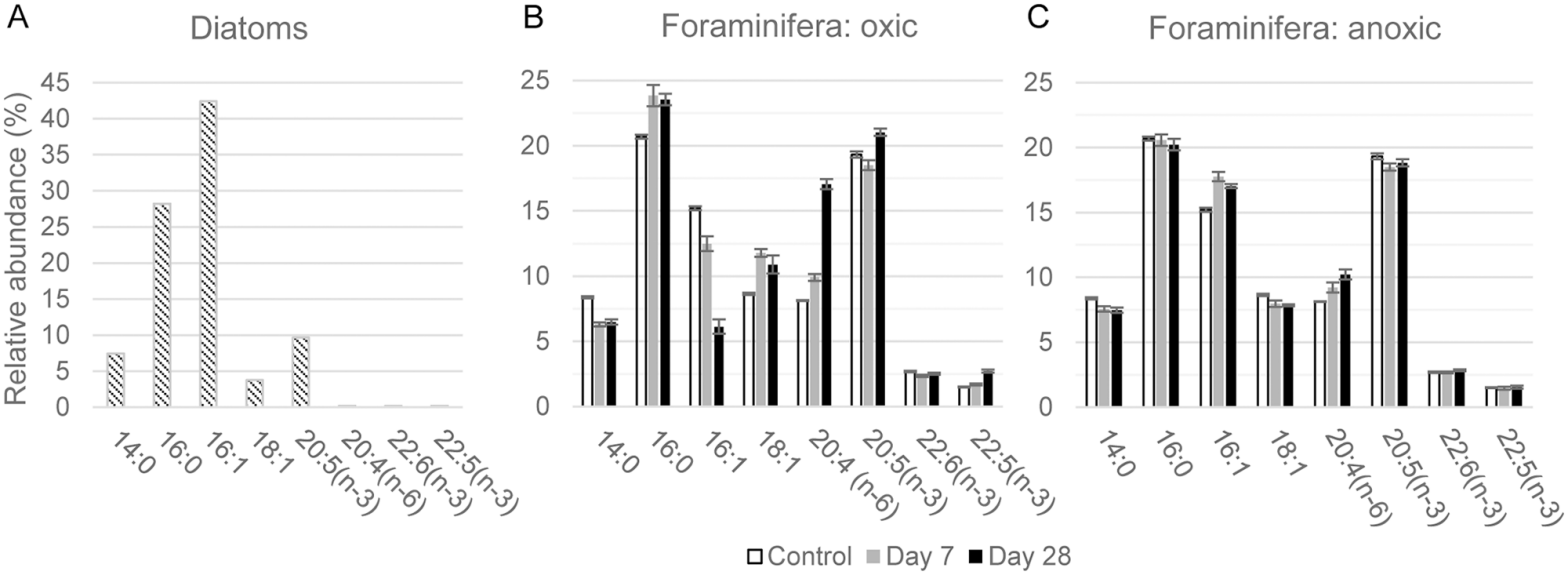
Relative abundances (%) of the dominant fatty acids extracted from the biofilm of diatoms and in *A*. *tepida endoplasm*. Relative abundances (expressed in %) of the eight dominant FAs extracted from the biofilm of diatoms and in *A*. *tepida* individuals incubated under oxic (B) and anoxic (C) conditions, respectively. White: control specimens; grey: after Day 7 and black: after Day 28. Error bars are ±1 SD (*n* = 3).

Fatty acids (FAs) studied here included triglycerides, phospholipid and free acids, as well as other acid lipids extracted from diatom and foraminifera samples. In the following, FAs are abbreviated as x:y(z) where ‘x’ is the number of carbon atoms, ‘y’ the number of double bonds and ‘z’ the position of the double bond relative to the terminal methyl group. The main saturated FAs in the labeled diatom biofilm were 14:0 and 16:0, with relative abundances of 7.4% and 28.2%, respectively (Fig 5A). The mono-unsaturated FAs 16:1 and 18:1 (isomers) were observed in relative abundances of 42.0 and 3.8%, respectively (Fig 5A). The sums of all the positional (mainly *n*-9, *n*-7and *n*-5) and geometric (*cis* and *trans*) isomers of hexadecenoic and octadecenoic acid were included in the designations 16:1 and 18:1. The main polyunsaturated FAs (PUFA) were 20:5(*n*-3) (9.6%) with trace amount of 20:4(*n*-6) and 22:5(*n*-3) and 22:6(*n*-3), which accounted for less than 0.2% of the total FAs.

All analyzed foraminifera samples showed roughly similar FAs distributions in the C_14_ to C_22_ range. The observed small quantities of odd-chain and traces or complete absence of branched-chain FAs indicate minimal bacterial contamination. In the control foraminifera, the most abundant saturated FAs were 14:0, 16:0 and 18:0, with a preference for 16:0 (Table 2 and Fig 5B and 5C). The most abundant monounsaturated FA was 16:1 and the most abundant PUFAs were 20:4(*n*-6) and 20:5(*n*-3) (Table 2 and Fig 5).

**Table 2:** Concentrations of fatty acids in *A*. *tepida*.

Fatty acid	Control	Oxic		Anoxic	
		7 days	28 days	7 days	28 days
Total	322.6±22.4	408.3±33.5	344.24±31.8	360.1±22.1	380.8±13.1
14:0	27.0±1.8	25.7±1.5	22.3±2.4	27.2±0.9	28.4±0.8
15:0	4.8±0.2	4.1±0.3	3.4±0.2	4.9±0.2	5.2±0.4
15:1		1.5±0.2	2.2±0.4		
16:0	66.7±4.3	97.3±7.4	81.1±8.2	74.1±5.7	77.0±3.4
16:1	49.1±3.8	51.1±6.4	21.2±3.5	64.0±5.1	64.9±2.7
16:2	11.4±0.8	9.3±3.3	3.2±2.7	14.0±0.4	14.7±0.4
16:3	3.1±0.2	2.9±0.5		2.9±0.3	3.3±0.5
17:0	3.3±0.4	1.1±0.3		3.4±0.2	3.6±0.1
17:1	3.2±0.4	3.7±0.3	2.8±0.2	2.1±1.3	2.3±1.5
18:0	8.6±0.4	14.6±1.5	11.3±0.5	8.3±1.5	8.7±0.4
18:1	27.9±2.2	48.1±4.95	37.6±5.5	28.7±2.5	22.9±1.3
18:2(*n*-6)	5.2±0.6	6.4±0.6	3.4±0.4	5.3±0.4	5.6±0.4
18:4(*n*-3)	3.4±0.3	3.1±0.2	2.2	4.3±0.3	4.7±0.1
20:1(*n*-9)	4.1±0.2	6.2±0.6	4.6±0.2	3.9±0.7	4.3±0.1
20:4(*n*-3)	2.6±0.2	2.6±0.2	3.0±0.1	2.9±0.2	2.9±0.2
20:4(*n*-6)	26.2±1.8	40.4±2.5	58.7±5.0	33.2±2.5	39.0±2.0
20:5(*n*-3)	62.3±4.3	75.6±6.3	72.5±7.5	66.6±4.3	71.7±3.1
22:6(*n*-3)	8.7±0.7	9.6±0.7	8.7±0,7	9.7±0.4	10.8±0.2
22:5(*n*-3)	4.8±0.4	6.9±0.3	9.3±0.6	5.3±0.3	5.9±0.3

Concentrations in ng×ind^-1^ of the fatty acids found in *A*. *tepida* cell before the experiment (control), after Day 7 and Day 28; under oxic and anoxic conditions.

Under oxic conditions, the FA content in foraminifera increased during the first 7 days from 322±22 to 408±33 ng×ind^-1^ (*p*<0.05), and then decreased to 344±32 ng×ind^-1^ after Day 28 (*p*<0.1 between 7 and 28 days) (Table 2). Under anoxia, the total foraminifera FA content continuously increased during the experiment from 322±22 up to 380±13 ng×ind^-1^ (*p*<0.05) (Table 2). Under oxic conditions, the relative abundances of 16:0 and 18:1 isomers increased between 0 and 7 days (*p*<0.05), and remained stable between Day 7 and 28 (Fig 5B). The relative abundances of 14:0 and 16:1(*n*-7) decreased between Days 0 (control) and 7 (*p*<0.05). Between Days 7 and 28, the relative abundance of 14:0 remained constant, while that of 16:1(*n*-7) continued to decrease. The abundance of 20:5(*n*-3) first decreased between Days 0 (control) and 7, and then increased to its highest level at Day 28 (*p*<0.05) (Fig 5B). Despite being present in small amounts in the diatom biofilm, the PUFAs 20:4(*n*-6) and 22:5(*n*-3) significantly increased in relative abundance along the experiment (*p*<0.05); most pronounced for 20:4(*n*-6) from 8.1% in the control to 17.1% (Fig 5A–5B). Significant variation in the abundance of 22:6(*n*-3) was not observed during the experiment (*p*>0.05).

Under anoxia, the relative variations in the abundance of individual FAs with time were significantly smaller than those observed under oxic conditions (Fig 5C). Only the abundance of 14:0 decreased slightly during the experiment, with a trend similar to that observed under oxic conditions. No significant changes (*p*>0.05) were observed in the contents of 16:0, 22:5(*n*-3), and 22:6(*n*-3) during the experiment. 16:1(*n*-7) first increased slightly, then decreased from Day 7 to Day 28 (*p*<0.05). 18:1 abundance first decreased at Day 7 (*p*<0.05), and stabilized (*p*>0.05). 20:4(*n*-6) was the only FA that showed a significant, albeit minor increase (from 8.1±0.1 to 10.2±0.4%; *p*<0.05) along the experiment.

The ^13^C atomic fraction of FAs (*x*(^13^C)_FA_ in %) are shown in Table 3. These ^13^C-enrichments were significantly higher after Day 7 of incubation (*p*<0.05) under both conditions and in general FAs ^13^C-enrichments were higher under oxic than anoxic conditions.

**Table 3:** ^13^C atomic fraction of dominant fatty acids in the cytoplasm of *A*. *tepida*.

Fatty acid	Control	Oxic		Anoxic	
		Day 7	Day 28	Day 7	Day 28
14:0	1.08±0.01	1.81±0.04	1.89±0.02	1.32±0.07	1.37±0.02
16:0	1.08±0.01	2.07±0.07	2.01±0.11	1.85±0.06	1.92±0.04
16:1	1.08±0.01	2.25±0.04	2.23±0.04	1.40±0.06	1.41±0.02
18:1	1.08±0.01	2.31±0.01	2.39±0.05	1.47±0.10	1.23±0.01
20:4(*n*-6)	1.08±0.01	1.77±0.04	1.93±0.04	1.28±0.05	1.33±0.02
20:5(*n*-3)	1.08±0.01	2.01±0.04	2.03±0.05	1.33±0.01	1.39±0.02
22:6(*n*-3)	1.08±0.01	1.69±0.03	1.72±0.01	1.22±0.01	1.31±0.02
22:5(*n*-3)	1.08±0.01	1.74±0.03	1.85±0.01	1.15±0.01	1.18±0.01

^13^C atomic fraction (*x*(^13^C) in %) of dominant fatty acids in the cytoplasm of *A*. *tepida* (*n* = 2) for oxic and anoxic conditions at Days 7 and 28.

## Discussion

### Survival and growth

No significant difference was observed between the survival rate of fed *A*. *tepida* specimens incubated for 13 days under oxic and anoxic conditions (S1 Fig). This is in line with results of previous laboratory experiments showing that *A*. *tepida* is capable of surviving under strong hypoxia and anoxia for extended time periods, up to 60 days [31,56]. Growth of *A*. *tepida* under anoxia was assessed by three different methods: (*i*) measurement of juvenile shell size before and after incubation (S2 Fig), (*ii*) quantification of the carbonate content in shells of adult specimens, and (*iii*) shell ^13^C-enrichment (S1 Table). The results consistently showed that on average *A*. *tepida* grew and added at least one chamber under oxic conditions, whereas only minimal, if any growth took place under anoxia. A previous study using incubation with calcein labeled foraminifera to detect chamber formation showed that among adult *A*. *tepida* living under anoxic conditions for 60 days, only 5% were able to add one chamber [31]. These observations clearly indicate that a strong perturbation of normal physiological processes results from a shift to anoxia.

### Feeding and metabolism: Bulk data

Under oxic conditions, the TOC and its ^13^C-fraction increased by almost 100%, within the first 7 days in fed, adult *A*. *tepida* (Fig 1). Similar TOC values are reported in the literature quantifying the rate of ingestion of diatoms [50], indicating an important role for benthic foraminifera in the organic carbon cycle in shallow, O_2_-rich marine sediments. After Day 7 under oxic conditions, the TOC began to decrease steadily while its ^13^C-enrichment remained constant (Fig 1), consistent with the visual observation that the food source had been exhausted. From this point onward, the TOC values decreased (*i*.*e*. cells lost weight), as their reserves of organic C (mainly in the form of lipid droplets) were metabolized and respired. This metabolic consumption of organic matter did not change the ^13^C content of the residual TOC, indicating that no preferential respiration or preservation of organic compounds with different isotopic composition took place.

Under anoxia *A*. *tepida* ingested ^13^C-enriched diatom biofilm only during Day 1. During this time, the ^13^C-enrichment of the TOC increased by about 30% and the average of TOC content per cell increased from the control value of 0.65 μg C×ind^-1^ to about 0.9 μg C×ind^-1^ (Fig 1). In contrast to the results from the corresponding oxic experiment, neither the TOC nor its ^13^C-enrichment changed substantially after Day 1, consistent with the visual observation that feeding stopped, *i*.*e*. left over biofilm was not further consumed. Furthermore, the TOC per cell did not decrease (Fig 1), providing strong indication that metabolic loss of carbon was minimal after Day 1.

Recent studies with ingestion of phytodetritus under strong hypoxia (O_2_ levels around 0.02 mL/L) have documented both ingestion and metabolism in species from the Arabian Sea oxygen minimum zones (OMZ) [48,49]. The fact that foraminifera metabolism seems relative insensitive to hypoxic conditions might be due to their low rate of oxic respiration compared to other benthic meiofauna [32]. A picture emerges of benthic foraminifera capable of maintaining an efficient metabolism even under strong hypoxia, while complete anoxia leads to a shutdown of aerobic metabolic processes on a timescale of less than 24 hours.

### Feeding and metabolism: Subcellular observations

Key sub-cellular structures of *A*. *tepida* involved in ingestion and metabolism include the ingested diatoms, residual bodies, and lipid droplets (S3 Fig), the latter representing the principal form of carbon storage [57–59]. Fully intact diatoms (*i*.*e*. with the diatom cell-material still contained in its silica frustule) were directly integrated into the cytoplasm by the foraminifera during the first day under both conditions (Figs 2A–2C and 3A–3C), consistent with previous observations of feeding *A*. *tepida* [60] and a number of other species [60–62]. Nevertheless, the density in the cytoplasm of ingested diatoms observed in our study was substantially higher than previously reported in the literature, with ca. 30% of the cytoplasm area occupied by intact diatoms after Day 1 under both conditions. This might be ascribed to the fact that the foraminifera had been fasting during the 6 days between the initial collection on the mudflat and the start of the feeding experiment, thus they grazed quickly on the available biofilm at the beginning of the incubation.

Following the efficient ingestion of intact diatoms during Day 1, the sub-cellular TEM and NanoSIMS observations for oxic and anoxic conditions diverged dramatically (Figs 2 and 3). Under oxic conditions, the intact diatom frustules were all emptied and their ^13^C-enriched contents incorporated into other subcellular components before Day 7. On Day 7, the silica frustules had almost entirely disappeared (Figs 2G and 4A). The process by which the foraminifera break down the frustules remains unknown. Exocytosis of the empty frustules was not observed, nor frustules being degraded.

Part of the organic diatomic material was converted into fatty acids stored in clearly ^13^C-labeled lipid droplets (Figs 2G–2I, 4C and 4D). After Day 7, the ^13^C-labeled diatomic material had become part of the metabolic pathways and ^13^C-enrichment had spread into most components of the cytoplasm (Fig 2I, 2L, 2O). Consistent with the observed decrease in TOC after Day 7 (Fig 1A), once the entire diatom biofilm had been ingested, the foraminifera began to metabolize their lipid reserves. As a result, lipid droplets had disappeared at Day 28 (Fig 4C). In contrast, residual bodies with clear ^13^C-enrichment appeared in the cytoplasm at Day 14 (Figs 2J, 4E and 4F). These heterogeneous vacuoles are believed to hold metabolic waste and recycled organelles [58,63]. The rapid ingestion, catabolism, and anabolism of the ^13^C-enriched diatom biofilm in *A*. *tepida* under oxic conditions (Fig 2) is consistent with the bulk observations discussed above and the evolution of the fatty acid composition discussed below. The observation of labeled organic lining in 5 of the foraminifera incubated in oxic conditions is likely to be linked with chamber formation, because the organic lining is thought to play a key role in initiating calcite formation [58,64]. Consistent with this, no ^13^C-labeled organic lining was observed in the specimens from anoxic conditions, which did not grow new chambers.

Under anoxic conditions, the metabolism was very different (Fig 3). Following the initial ingestion of diatoms during Day 1, there was substantially less redistribution of ^13^C-enriched material in the foraminifera cells until the experiment ended. On Day 28, diatoms with their frustules were still present in the cytoplasm with their original content of strongly ^13^C-labeled material (Fig 3M–3O). Nevertheless, some early metabolism/redistribution did occur, resulting e.g. in the appearance of ^13^C-enriched lipid droplets from Day 3 (Fig 4C). The density of lipid droplets remained constant after Day 3, consistent with the observation of constant average TOC levels (Fig 1A). The formation of lipid droplets earlier in anoxic (Day 3) than in oxic conditions (Day 7) might be attributed to the stressful conditions: faced with a lack of oxygen the foraminifera were first storing carbon in lipid droplets instead of using it for the cell metabolism. A qualitatively similar increase of lipid droplet abundance was observed in *Ammonia beccarii* specimens submitted to stress from Cu contamination [65]. Such a response does not seem to be specific to foraminifera; it has also been observed in marine dinoflagellates [66]. Mildly ^13^C-enriched residual bodies did not appear until between Day 21 and 28 (Figs 3M–3O and 4E–4F).

### Fatty acid composition and synthesis

Fatty acids 14:0, 16:0, 16-1(*n*-7), and specifically the 20:5(*n*-3) (Fig 5A, Table 2) are biomarkers of marine diatoms [67–69]. These FAs had already been observed in algae feeding experiments with foraminifera under both oxic [70] and hypoxic conditions [49,52]. In our study, the observed increase during the first 7 days of 16:0 under oxic conditions and of 16:1(*n*-7) under anoxic conditions is ascribed to the ingestion of diatoms. The decreases of 14:0 under both conditions and of 16:1(*n*-7) under oxic conditions at Day 7 suggest lipolysis and fatty acid catabolism (their β-oxidation to C_2_ units). Part of the degradation products were probably used for *de novo* synthesis of long chain fatty acid intermediates for the production of PUFAs, *i*.*e*. 20:4(*n*-6), 20:5(*n*-3), and 22:5(*n*-3) under oxic conditions (Fig 5B). Under oxic conditions, the relative abundance of 20:5(*n*-3) first decreased and then increased. This suggests that this PUFA was first consumed (by metabolic breakdown or used for the synthesis of 22:5) and then formed by desaturation and C_2_ elongation of short-chain precursors. The relative increase in 20:5(*n*-3) cannot be explained by an ingestion of diatoms, because there were completely ingested after Day 7 under oxic conditions. This supports *de novo* synthesis of eicosapentaenoic acid 20:5(*n*-3) by the foraminifera.

20:4(*n*-6) and 22:5(*n*-3) were present only in small abundances in the diatom biofilm (Fig 5A), but in higher concentrations in *A*. *tepida* cytoplasm under both oxic and anoxic conditions (Fig 5B and 5C and Table 2). This can be explained by either a selective uptake of these PUFAs [70], or by *de novo* biosynthesis following a pathway similar to that for 20:5(*n*-3). A similar high increase in 20:4(*n*-6) content was observed in other foraminiferal feeding experiment with microalgae [49,71–73]. The observed concentration increase, combined with significant ^13^C-enrichment (Fig 5B and 5C and Table 2), strongly suggest *de novo* synthesis of this arachidonic acid, as hypothesized in other publications [49,73].

*A*. *tepida* is also able to graze on bacteria [55]. The increase in the relative abundance of 18:1, which is a bacterial biomarker in marine environments [74], during the first 7 days under oxic conditions (Fig 5B) suggests that bacteria developed during the beginning of the experiment, assimilating ^13^C by degrading the de-frozen diatom biofilm (Fig 5B and Table 3). Further support for ingestion of bacteria is provided by the presence of small amounts of other bacterial FAs (15:0, 15:1, 17:0, and 17:1) in the foraminiferal cells (Table 2).

Finally, under anoxic conditions, the foraminifera assimilated clearly less ^13^C labeled fatty acids from the diatom biofilm than under oxic conditions (Table 3, Fig 5B and 5C), and they produced less new fatty acids. Between Days 7 and 28, only the relative abundances of the FAs 16:1(*n*-7) and 20:4(*n*-6) varied, indicating some, albeit strongly reduced metabolism compared to oxic conditions.

Together, our observations under anoxia indicate that food digestion and metabolic redistribution took place at a much-reduced rate compared to oxic conditions. Nevertheless, anabolic processes did initially take place, conceivably driven by the ‘oxic metabolic machinery’ still available to the cell during the first hours after establishment of anoxic conditions. The reduced state of metabolism seems consistent with a state of dormancy or quiescence, defined as a suspension of active life, arrested development, and reduced or suspended metabolic activity [42], in our case due to the sudden onset of anoxic conditions. Consistent with a state of dormancy/quiescence is the fact that no obvious ultrastructural damage to the cells was observed, indicating that capability to return to a state of normal vitality once oxic conditions are reestablished.

## Conclusions

Benthic foraminifera *Ammonia tepida* are ubiquitous in coastal marine sediments, where they are often exposed to hypoxia or completely anoxic conditions. In order to survive such anoxic conditions for longer time periods they must either rely on alternative, anaerobic metabolism, which would allow them to produce energy and thus maintain a certain level of activity, or enter a state of dormancy that minimizes energy consumption. With a broad suite of observations we show here that these single cell organisms respond to anoxic conditions by a radical reduction in their heterotrophic metabolism. This, combined with the observation of arrested calcification and the complete absence of physical movements upon exposure to anoxia (movement is restored when oxygen is returned to the environment [75]), indicates that these species do not have access to an alternative metabolic mechanism allowing them to maintain, even approximately, their level of physical activity under oxic conditions. Therefore, we propose that, upon exposure to anoxia, the *A*. *tepida* organism enters into a state of dormancy/quiescence, with strongly reduced metabolic requirements that make them capable of withstanding anoxic conditions for unusually long time intervals (here up to 28 days), compared with other benthic meiofauna.

## Material and methods

### Experiment I: Survival and growth rate of *A*. *tepida*

Superficial (top 2 cm) sediment was collected at low tide on January 15, 2013, from the intertidal mudflat of l’Aiguillon Bay (France). Living foraminifera were picked out of sieved sediment of two size fractions: >150 μm (adults) and 100–150 μm (juveniles).

Experiment I took place at the LPG-BIAF laboratory (Angers, France). For determination of the survival rate 300 adult foraminifera were checked for their vitality using 2 criteria: presence of yellow brownish cytoplasm in the shell and detection of movement of the foraminifera [56]. For determination of growth rate, 150 juveniles at a growth stage with 8 ±1 chambers were selected using the same criteria as for adults.

Incubation was carried out in two glass aquaria (33×21×19 cm^3^) containing 10 liters ASW (RedSea Salt, salinity of 35 psu), under oxic and anoxic conditions, respectively. Each aquarium contained eighteen 10 mL glass vials (h = 45 mm, ø = 22 mm), 15 vials holding 10 adult individuals and 3 vials holding 25 juvenile individuals, with each vial representing a replicate in subsequent calculations. Before the start of the experiment, a thin layer of freeze-dried *Chlorella* algae was added, forming a biofilm on the vial bottom (14.3 μg chlorophyll×cm^-2^). Each vial was then covered with a 100 μm mesh net, the aquaria were covered with Plexiglas lids to minimize evaporation and avoid changes in salinity and the lid of the anoxic aquarium was sealed with plastic tape to prevent gas leakage/exchange. Each aquarium was bubbled continuously with air using a standard aquarium pump to maintain oxic conditions, or with a mixture of N_2_ and 0.04% CO_2_ (Air liquide, France, 99.999% N_2_, 99.99% CO_2_) to produce anoxic conditions. Bubbling began immediately after the foraminifera were placed inside. The incubation started on the 12^th^ of February 2013 and lasted 13 days. Oxygen concentrations, temperature, salinity and pH were measured continuously (oxygen and temperature) or at the beginning and end of the experiment (salinity and pH) using dedicated sensors (details in S2 Table). O_2_ contents were between 4.0 to 4.5 mL×L^-1^, and below 0.007 mL×L^-1^ (detection limit) in the oxic and anoxic aquaria, respectively. Temperature was between 17.5 and 19.5°C, salinity 35.2±0.2 psu and pH 8.1±0.1. After 13 days, the incubation was stopped and the vials with the foraminifera taken out of the aquaria.

To determine survival rates, individuals were immediately incubated with 10 μM FDA (fluorescein diacetate) solution [56]. After rinsing, fluorescence of the foraminifera was immediately observed with an epifluorescence stereomicroscope (Olympus SZX16, LPG-BIAF laboratory) equipped with a fluorescent light source (Olympus U-RFL-T). Foraminifera with less than 3 chambers not fluorescing (terminal chambers) were considered to be alive. The average size of all juveniles was measured before (*t*_0_) and after (*t*_1_) the incubation using an automatic particle analyzer (LPG-BIAF laboratory) equipped with an automated incident light microscope system; a Leica CLS100X ring light source mounted on a monocular Leica Z16PO microscope. A camera (SIS CC12) recorded images and the size of individuals was determined with the software analySIS FIVE (SIS/Olympus) [76]. The growth rate (in % size change) was calculated as: $\frac{Size\;t1-Size\;t0}{Size\;t0}\times 100$.

### Experiment II: Feeding behavior of *A*. *tepida* under oxic and anoxic conditions

Superficial (top 2 cm) sediment was collected at low tide on March 27, 2014, on the intertidal mudflat of the Bay of Bourgneuf (France). Living foraminifera were picked out from sieved sediment and transported to LGB laboratory (EPFL, Switzerland).

The diatom *Navicula salinicola* (CCAP, strain 1050/10) was grown for one week in F2 medium enriched with 2 mM of ^13^C-enriched sodium bicarbonate (^13^C fraction of 99%, Sigma-Aldrich, Switzerland). The F2 medium was made with non-decarbonated water with an original concentration of ~2 mM sodium bicarbonate. Thus the addition of 2 mM of ^13^C-enriched sodium bicarbonate resulted in a labeling of roughly 50% of the dissolved inorganic carbon (DIC). The microalgae were harvested by centrifugation (1500 g, 10 min), washed 3 times with artificial seawater (RedSea Salt, salinity of 35 psu) to remove the excess NaH^13^CO_3_, and frozen at –20°C until use in the experiment.

Starting on April 2^nd^, 2014 (six days after collection on the mudflat and one day before the feeding experiment began), living *A*. *tepida* specimens were selected under a binocular microscope, with the same criteria as in Experiment I. A total of about 6000 individuals were distributed in 93 10 mL glass vials (h = 45 mm, ø = 22 mm), so that each vial contained ca. 65 specimens. 39 vials with foraminifera were placed in each aquarium. Fifteen vials containing foraminifera were used as control material for the subsequent analyses: 3 for TEM-NanoSIMS and total organic carbon (TOC) quantification and stable isotope analysis; 12 for fatty acid analysis. These were placed overnight in ASW (RedSea Salt, salinity of 35 psu) under oxic conditions without feeding and were sampled on Day 1, *i*.*e*. during the first sampling of foraminifera.

Incubation was performed as in Experiment I in oxic and anoxic aquarium. After 4 hours of bubbling with the mixture of N_2_ and 0.04% CO_2_ (Carbagas AG, Switzerland), enough to allow the complete depletion of O_2_ in the anoxic aquarium, the experiment started. All the foraminifera were fed by adding ^13^C-enriched diatoms (ca. 578 mg C×m^-2^) to all vials (*i*.*e*. in both oxic and anoxic aquaria) over a timespan of a few minutes. Anoxic and oxic conditions, were maintained from this point onwards. Oxygen concentrations were in the range of 4.1–4.8 mL×L^-1^ and below 0.007 mL×L^-1^ in the oxic and anoxic aquaria, respectively. Throughout the experiment, temperature was between 23 and 24°C, salinity 32 psu and pH 8.3.

For TEM-NanoSIMS and TOC quantification and stable isotope analysis, 3 vials were harvested at 1, 3, 7, 14, and 28 days from each aquarium. In addition, 12 vials were harvested from each aquarium for fatty acid analyses at Day 7 and Day 28, respectively. Immediately upon removal from the aquaria, the foraminifera were incubated for 3 h at room temperature in the dark with FDA to a concentration of 100 μM [77]. Vitality was assessed under an epifluorescent stereomicroscope (Leica M165C equipped with SFL100 LED fluorescence module; GFP green). Only living specimens were selected for further analysis. After rinsing with ASW, individuals for TEM-NanoSIMS analysis were immediately processed, those for TOC and fatty acid analysis were stored in cleaned and pre-heated 5 mL glass vials at –20°C until required.

#### TEM and NanoSIMS analysis

After incubation with FDA, specimens were immediately fixed and prepared for TEM imaging using standard procedures (details can be found in S1 Text) and observed with a transmission electron microscope (TEM, Philips 301 CM100, 80 kV) at the Electron Microscopy Platform of the University of Lausanne. Ultra-thin sections observed with TEM were subsequently imaged with a NanoSIMS ion microprobe [78]. Areas of interest for NanoSIMS imaging were selected based on TEM observations permitting direct correlation of ultrastructural (TEM) and isotopic images. Our observations systematically focused on the antepenultimate chamber of the foraminifera, i.e. the third chamber counting from the aperture. NanoSIMS imaging followed established procedures [79–81], as detailed in S1 Text. Regions of interest (ROIs) were drawn with the software Look@NanoSIMS [82] to estimate the percentage of cytoplasmic occupation and to quantify mean ^13^C enrichments of different sub-cellular structures of a given foraminifera. ^13^C enrichments were reported as ^13^C atom fraction in %: x(^13^C) = ^13^C/(^13^C+^12^C)×100.

#### Fatty acids

Foraminifera from oxic and anoxic conditions sampled at Days 7 and 28, respectively, plus a sample of the ^13^C-labeled diatomic biofilm were analyzed for their fatty acids (FAs) composition using procedures adapted from [83]. Each sample was analyzed in triplicate, and the mean value was used for further calculations. For each analysis, lipids were extracted from 200 water-washed and dried specimens by sonication with mixture of methanol and dichloromethane of decreasing polarity. An aliquot of internal standard solution of deuterated carboxylic acids was added to permit quantification. The carboxylic acids were obtained by alkaline hydrolysis of the organic extract and were methylated with BF_3_/MeOH to obtain fatty acid methyl esters (FAMEs). Chemical characterization of the fatty acids (as FAMEs) was performed by gas chromatography/mass spectrometry and quantification by gas chromatography/flame ionization detection (details in S2 Text).

#### Stable isotope analyses by isotope ratio mass spectrometry (IRMS)

Compound specific stable C isotopic composition of fatty acids was measured by gas chromatography/combustion/isotope ratio mass spectrometry. The standard deviation for repeatability of the ^13^C atomic fraction, *x*(^13^C)_FA_ in %, ranged between ±0.01% and ±0.06%. The lipid-free foraminifera carbonate shell were analyzed for their ^13^C atomic fraction, *x*(^13^C)_car_, using a carbonate preparation device (GasBench II, Thermo Fisher Scientific, Bremen, Germany) and isotope ratio mass spectrometry. The measured shell ^13^C atom fractions, *x*(^13^C)_car_, had a precision of ±0.01% (2 SD). The average carbonate content (in μg C×ind^-1^) of the shells was determined from the peak area of the major ions, ±0.02 μg C×ind^-1^ for TOC content. The ^13^C atom fraction of the total organic matter, *x*(^13^C)_TOC_, of decalcified foraminifera were determined by continuous flow elemental analysis/isotope ratio mass spectrometry. For each analysis, 30 previously decalcified specimens were used. The total organic carbon (TOC) content was determined from the peak area of the major isotopes and expressed in microgram per individual cell (μg C×ind^-1^). Reproducibility and accuracy were better than ±0.01% for *x*(^13^C)_TOC_ (2 SD) and ±0.02 μg C×ind^-1^ for TOC content. For each analysis, 30 specimens were used (details in S3 Text).

#### Statistical analysis

Data were analyzed using the R software. Univariate ANOVA tests were performed to compare the effects of the time and experimental conditions (*i*.*e*. oxic *vs*. anoxic). To determine the significance between two time points or two conditions at the same time point, the Tukey *post-hoc* test was carried out following the ANOVA. For the fatty acid abundance data, two-sample *t*-tests were performed to investigate significance of variations between time points for a given condition. Variances of the data were checked with a *F*-test prior the *t*-tests. The used significance level for all the tests was *α* = 0.05.

Access to both sampling sites did not required any specific permissions, and the work did not involve endangered or protected species

## Supporting information

S1 Fig Survival rate Experiment I. (DOCX)

S2 Fig Growth rate Experiment I. (DOCX)

S3 Fig Typical cellular structures of Ammonia tepida cytoplasm. (DOCX)

S1 Table Carbonate uptake and shell ^13^C-enrichment. (DOCX)

S2 Table Sensors used in Experiment I and II. (DOCX)

S1 Text TEM and NanoSIMS imaging. (DOCX)

S2 Text Detailed protocol of fatty acid analysis. (DOCX)

S3 Text Detailed protocol of stable isotope analyses by isotope ratio mass spectrometry (IRMS). (DOCX)
