# Supplementary material for: Surviving anoxia in marine sediments: The metabolic response of ubiquitous benthic foraminifera (Ammonia tepida)
Source: PLoS One. 2017 May 31;12(5):e0177604. doi: 10.1371/journal.pone.0177604 (PMC5451005; doi:10.1371/journal.pone.0177604)
Supplement: S1 Fig — (DOCX) [file pone.0177604.s001.docx]

S1 Figure: Survival rate Experiment I

Survival rate (%) of adults (striped bars) and juveniles (solid bars) of *A. tepida* incubated for 13 days in oxic and anoxic conditions (*n*=10). Error bars are ±1 SD.
