# Supplementary material for: Surviving anoxia in marine sediments: The metabolic response of ubiquitous benthic foraminifera (Ammonia tepida)
Source: PLoS One. 2017 May 31;12(5):e0177604. doi: 10.1371/journal.pone.0177604 (PMC5451005; doi:10.1371/journal.pone.0177604)
Supplement: S2 Fig — (DOCX) [file pone.0177604.s002.docx]

S2 Figure: Growth rate Experiment I

Growth rate (%) of juveniles incubated 13 days in oxic and anoxic condition (n=3). Error bars are ±1 SD.
