# Supplementary material for: Surviving anoxia in marine sediments: The metabolic response of ubiquitous benthic foraminifera (Ammonia tepida)
Source: PLoS One. 2017 May 31;12(5):e0177604. doi: 10.1371/journal.pone.0177604 (PMC5451005; doi:10.1371/journal.pone.0177604)
Supplement: S3 Fig — (DOCX) [file pone.0177604.s003.docx]

S3 Figure: Typical cellular structures of *Ammonia tepida* cytoplasm


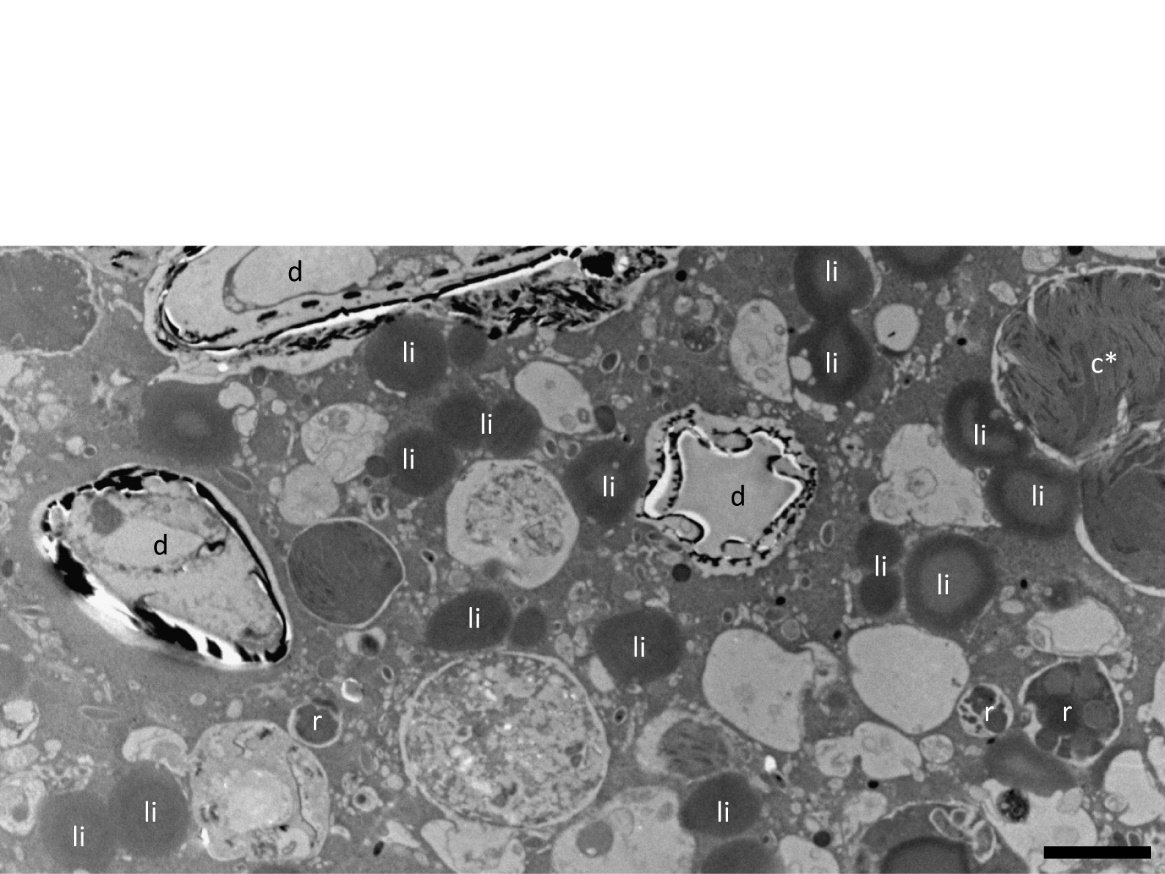


TEM image of the cytoplasm structures in the antepenultimate chamber of an *A. tepida* specimen collected *in situ*. Lipid droplets (li) are dark, homogenous vesicles with diameters between 1 to 5µm, no visible membrane surround them. Residual bodies (r) are circular heterogeneous vacuoles with diameters of about 1 to 5 µm and heterogeneous content. Empty diatomic frustules (d) and a chloroplast in degradation (c*) are visible. Scale bar: 2 µm.
