# Supplementary material for: Surviving anoxia in marine sediments: The metabolic response of ubiquitous benthic foraminifera (Ammonia tepida)
Source: PLoS One. 2017 May 31;12(5):e0177604. doi: 10.1371/journal.pone.0177604 (PMC5451005; doi:10.1371/journal.pone.0177604)
Supplement: S1 Table — (DOCX) [file pone.0177604.s004.docx]

S1 Table: Carbonate uptake and shell ^13^C-enrichment

|  | Oxic | Anoxic |
| --- | --- | --- |
| Carbonate uptake (µg C_car_×ind^-1^) | 4.9±1.8 | 0.2±0.4 |
| Δ^13^C=(*x*(^13^C_car_)_sample_ – *x*(^13^C)_car_) _control_ (%) | 0.05±0.01 | 0.00±0.01 |

Carbonate uptake (calculated as the difference of the C_car_ content of the foraminiferal shells of the individuals collected after 28 days and the foraminiferal shells of the control individuals, in µg C_car_×ind^-1^) and ^13^C enrichment (calculated as the difference of the *x*(^13^C)_car_ values of the foraminiferal shells of the individuals collected after 28 days and the foraminiferal shells of the control individuals, in %) of *A. tepida* carbonate shells after 28 days under oxic and anoxic conditions with feeding (*n*=3).
