# Supplementary material for: Surviving anoxia in marine sediments: The metabolic response of ubiquitous benthic foraminifera (Ammonia tepida)
Source: PLoS One. 2017 May 31;12(5):e0177604. doi: 10.1371/journal.pone.0177604 (PMC5451005; doi:10.1371/journal.pone.0177604)
Supplement: S2 Table — (DOCX) [file pone.0177604.s005.docx]

S2 Table: Sensors used in Experiment I and II

| Parameter | Experiment I | Experiment II |
| --- | --- | --- |
| Oxygen concentration | Continuously recorded:  OX-100, Unisense, Denmark | Continuously recorded:  OXROB3 for oxic aquarium and TROXROB3 (PyroScience) for anoxic aquarium |
| Temperature | Continuously recorded:  testo 175 T1 | Continuously recorded:  **TSUB36,** PyroScience |
| pH | Measured at the beginning and the end of the experiment:  pH-500C, Unisense, Denmark | Measured at each sampling time point:  Portable pH meter [pH 3310](http://www.wtw.com/en/products/product-categories/portable-meters/portable-meters/profiline-ph-3310.html), WTW |
| Salinity | Measured at the beginning and the end of the experiment:  Handheld meter Cond 330i, WTW | Measured at each sampling time point:  Handheld meter Cond 330i, WTW |
