# Supplementary material for: Surviving anoxia in marine sediments: The metabolic response of ubiquitous benthic foraminifera (Ammonia tepida)
Source: PLoS One. 2017 May 31;12(5):e0177604. doi: 10.1371/journal.pone.0177604 (PMC5451005; doi:10.1371/journal.pone.0177604)
Supplement: S1 Text — (DOCX) [file pone.0177604.s006.docx]

S1 Text: TEM and NanoSIMS imaging:

After incubation with FDA, specimens were rinsed 3 times in ASW (RedSea Salt, salinity: 35 psu), then chemically fixed as follows: fixation for 24 h with a mix of 4 % glutaraldehyde and 2 % paraformaldehyde 0.2 M cacodylate buffer, 0.4 M sucrose and 0.1 M NaCl (pH 7.4, room temperature, then storage at 4 °C). After rinsing, specimens were first decalcified with a solution EDTA 0.1 M, then post-fixed in 2 % osmium tetroxide. After a series of dehydration steps in ethanol (50, 70, 95, and 100 %), samples were embedded into an acrylic resin (LR White Resin Hard Grade). Specimens were cut into 70 nm sections with an ultramicrotome (Reichert Ultracut S) using a diamond knife (Diatome, Ultra, 45°). Sections were placed on electron microscopy copper grids with a formvar-carbon film, then stained for 10 min with 2 % uranyl acetate, rinsed, and observed with a transmission electron microscope (TEM, Philips 301 CM100, 80 kV) at the Electron Microscopy Platform of the University of Lausanne. Ultra-thin sections observed with TEM were subsequently imaged with a NanoSIMS ion microprobe (Hoppe et al., 2013). Areas of interest for NanoSIMS imaging were selected based on TEM observations permitting direct correlation of ultrastructural (TEM) and isotopic images. Our observations systematically focused on the antepenultimate chamber of the foraminifera, *i.e.* the third chamber counting from the aperture.

Prior to NanoSIMS imaging, the TEM grids were coated with 10 nm of gold to prevent charging effects. Images of typically 30×30 µm^2^ with 256×256 pixels were obtained by rastering a 16 keV primary Cs^+^ beam (about 2 pA) focused to a size of 120-150 nm across the sample surface with a dwell-time of 5 milliseconds. Secondary cyanide ions (^12^C^14^N- and ^13^C^14^N-) were simultaneously collected in electron multipliers at a mass resolution (M/ΔM) of about 9000 (Cameca definition), enough to resolve ^13^C^14^N^-^ from its ^12^C^15^N^-^ interference. Each NanoSIMS image consist of 6 sequential images, drift corrected, and accumulated using the software L’IMAGE (developed by Dr. Larry Nittler, Carnegie Institution of Washington, USA). Carbon isotope ratio images were obtained by taking the ratio between the cumulated ^13^C^14^N^-^ and ^12^C^14^N^-^ images.

Regions of interest (ROIs) were drawn with the software Look@NanoSIMS (Polerecky et al., 2012) to quantify mean ^13^C enrichments of different sub-cellular structures of a given foraminifera. The average ^13^C enrichment (and its standard deviation) for a given class of ROIs was obtained from images of 3 different foraminifera for each time point. The ROIs were also used to determine the percentage area occupied by a given type of structure in the cytoplasm (the sum of the pixels for a given type of ROI divided by the total number of pixels in the corresponding total images), providing an estimate of the abundance of a structure as a function of time.
