# Supplementary material for: Surviving anoxia in marine sediments: The metabolic response of ubiquitous benthic foraminifera (Ammonia tepida)
Source: PLoS One. 2017 May 31;12(5):e0177604. doi: 10.1371/journal.pone.0177604 (PMC5451005; doi:10.1371/journal.pone.0177604)
Supplement: S2 Text — (DOCX) [file pone.0177604.s007.docx]

S2 Text: Detailed protocol of fatty acid analysis

Foraminifera from oxic and anoxic conditions sampled at Days 7 and 28, respectively, plus a sample of the ^13^C-labeled diatomic biofilm were analyzed for their fatty acids (FAs) composition in the IDYST laboratory at the University of Lausanne using procedures adapted from Spangenberg et al. (2014). Extraction and separation of acid lipids was carried out on three replicates for each time point and on one sample for the biofilm. For each lipid analysis of foraminifera cells, 200 specimens were rinsed with water purified using a Millipore® Direct-Q 3 System (Millipore Corporation, Bedford, MA, USA) to remove attached material from the shell. This material was frozen at –20 °C for 2 days, freeze-dried for 1 day in a Lyovac GT2 freeze-dryer (SRK System Technik GmbH, Goddelau, Germany) and stored at –20 °C for lipid extraction. An aliquot of internal standard solution containing a defined amount of deuterated carboxylic acids (D_23_*n*-C_12:0_, lauric acid, D_39_*n*-C_20:0_, arachidic acid) was added to each sample, permitting identification and quantification. Lipids were then extracted using sonication in solvents of decreasing polarity (10 min in 2 mL methanol, 10 min in 2 mL methanol/dichloromethane, 1:1, v/v; 2×10 mins in 2 mL dichloromethane). The extracts were combined and the solvent removed via gentle evaporation under a clean nitrogen flow. The carboxylic acids were obtained by hydrolysis with 10 % KOH/MeOH at room temperature for 16 h. The non-saponifiables were separated with hexane. The fraction containing the acid lipids was acidified with 1 N HCl to pH<1, and the acid lipids extracted with hexane and methylated (boron trifluoride/methanol solution) to provide fatty acid methyl esters (FAMEs). The FAMEs were stored at 4 °C until analysis. Chemical characterization of FAMEs was performed by gas chromatography/mass spectrometry (GC/MS) using an Agilent (Palo Alto, USA) 6890 gas chromatograph connected to an Agilent 5973 mass selective detector operating at 70 eV (source 230 °C and quadrupole 150 °C) in the electron ionization mode with emission current 1 mA and multiple ion detection over m/z 45 to 750. Helium was used as carrier gas. The FAMEs were analyzed with two different fused silica columns and GC temperature programmed to permit the detection of long chain FAs and good separation of unsaturated FAs: (i) HP-ULTRA 2 (50 m × 0.32 mm; length × inner diameter) coated with 0.17 µm 5 % phenylmethylsilicone stationary phase. Samples were injected splitless at 320 °C. After an initial period of 2 min at 100 °C, the column was heated to 310 °C (held 20 min) at 4 °C/min. (ii) HP-FFAP (50 m × 0.20 mm; length × inner diameter) coated with 0.33 µm nitroterephthalic acid modified polyethylene glycol stationary phase. Samples were injected splitless at 200 °C. After an initial period of 2 min at 100 °C, the column was heated to 240 °C (held 30 min) at 5 °C/min. Compound assignment was based on comparison with standards, GC retention time, and MS fragmentation patterns. FAMEs were quantified by gas chromatography with flame ionization detection (GC/FID). An Agilent Technologies (Wilmington, USA) 7890B GC system equipped with a 7693A automated injection system and a flame ionization detector was used. Gas chromatography/flame ionization detection (GC/FID) analyses were performed using the HP-ULTRA 2 column and same chromatographic conditions as for GC/MS. The concentrations of the FAs were obtained from the GC/FID peak areas and expressed in nanogram per individual cell (ng×ind^-1^). One blank sample was run for every six samples throughout the analytical procedure. The absence of any measurable recovered extract from the blanks indicates that no detectable laboratory contamination was introduced to the foraminifera and biofilm samples during the analytical procedure.
