# Supplementary material for: Surviving anoxia in marine sediments: The metabolic response of ubiquitous benthic foraminifera (Ammonia tepida)
Source: PLoS One. 2017 May 31;12(5):e0177604. doi: 10.1371/journal.pone.0177604 (PMC5451005; doi:10.1371/journal.pone.0177604)
Supplement: S3 Text — (DOCX) [file pone.0177604.s008.docx]

S3 Text: Detailed protocol of stable isotope analyses by isotope ratio mass spectrometry (IRMS)

The stable carbon isotope analyses using IRMS were also performed in the Stable isotope laboratory at IDYST. For each time point, 3 replicates were analyzed, except in the case of stable C isotope analysis of individual fatty acids, for which 2 replicates were analyzed.

*C-isotope analysis of total organic carbon (TOC).* The ^13^C atom fraction, *x*(^13^C), of the total organic matter of decarbonated living foraminifera were determined by continuous flow elemental analysis/isotope ratio mass spectrometry (EA/IRMS) using flash combustion on a Carlo Erba 1108 elemental analyzer (Fisons Instruments, Milan, Italy) connected via a ConFlow III open split interface to a Delta V Plus isotope ratio mass spectrometer (Thermo Fisher Scientific, Bremen, Germany). For each analysis, 30 specimens were placed into a silver capsule, acidified with 1 drop of 10 vol. % HCl, let to react and dry for 16 h before EA/IRMS analysis. The total organic carbon (TOC) content was determined from the peak area of the major isotopes and expressed in microgram per individual cell (µg C×ind^-1^). Reproducibility and accuracy were better than ±0.01 % for *x*(^13^C)_TOC_ (2 SD) and ±0.02 µg C×ind^-1^ for TOC content.

*C-isotope analysis of the foraminiferal carbonate shells*. The foraminifera carbonate shell C-isotopic ratio, *x*(^13^C)_car_, was determined using a Thermo Fisher Scientific (Bremen, Germany) carbonate preparation device and GasBench II equipped with a Combi-Pal autosampler (CTC Analytics AG, Zwingen, Switzerland) and coupled to a Delta Plus XL isotope ratio mass spectrometer (both Thermo Fisher Scientific). We analyzed shells recovered after lipid-extraction for fatty acid analysis (see below). In each analysis, 10 lipid-free specimens were placed in a 12 mL vials (LABCO Ltd., Lampeter, UK) and immersed in 5 % sodium hypochlorite (NaOCl) solution for 12 h to remove potentially remaining labile organic compounds, washed with Millipore water, and dried at 40 °C. The vials were sealed with septum caps (from LABCO) and placed in an aluminum heating block at 70 °C for CO_2_ extraction by reaction with anhydrous phosphoric acid. The measured shell ^13^C atom fractions, *x*(^13^C)_car_, had a precision of ±0.01 % (2 SD). The average carbonate content (in µg C×ind^-1^) of the shells was determined from the peak area of the major ions, ±0.02 µg C×ind^-1^ for TOC content.

*Carbon isotope analysis of individual fatty acids*. Compound specific stable C isotopic composition of fatty acids in living foraminifera incubated under oxic and anoxic conditions, respectively, was measured by gas chromatography/combustion/isotope ratio mass spectrometry (GC/C/IRMS) using an Agilent 6890 GC instrument coupled to a Thermo Fisher Scientific (Bremen, Germany) Delta V Plus isotope ratio mass spectrometer via a combustion interface III under a continuous He flow. GC separation was performed with the same column and chromatographic condition as for GC/FID. Background subtracted sTable 1sotope compositions were first calculated using the Thermo Fisher Scientific ISODAT 2.5 software. For calibration were used the previously determined C isotopic compositions (by EA/IRMS) of the deuterated carboxylic acids added as internal standards. For quality control, the repeatability and intermediate precision of the GC/C/IRMS analysis and the performance of the GC and combustion interface were evaluated every 5 runs by injection of a carefully prepared mixture of FAMEs reference materials and duplicate analyses of the foraminifera samples FAME fractions. The standard deviation for repeatability of the ^13^C atomic fraction, *x*(^13^C)_FA_ in %, ranged between ±0.01 % and ±0.06 %.
